# Rebamipide Delivered by Brushite Cement Enhances Osteoblast and Macrophage Proliferation

**DOI:** 10.1371/journal.pone.0128324

**Published:** 2015-05-29

**Authors:** Michael Pujari-Palmer, Shiuli Pujari-Palmer, Håkan Engqvist, Marjam Karlsson Ott

**Affiliations:** Division of Applied Material Science, Department of Engineering Sciences, Uppsala University, Uppsala, Sweden; University of Texas Southwestern Medical Center, UNITED STATES

## Abstract

Many of the bioactive agents capable of stimulating osseous regeneration, such as bone morphogenetic protein-2 (BMP-2) or prostaglandin E2 (PGE2), are limited by rapid degradation, a short bioactive half-life at the target site in vivo, or are prohibitively expensive to obtain in large quantities. Rebamipide, an amino acid modified hydroxylquinoline, can alter the expression of key mediators of bone anabolism, cyclo-oxygenase 2 (COX-2), BMP-2 and vascular endothelial growth factor (VEGF), in diverse cell types such as mucosal and endothelial cells or chondrocytes. The present study investigates whether Rebamipide enhances proliferation and differentiation of osteoblasts when delivered from brushite cement. The reactive oxygen species (ROS) quenching ability of Rebampide was tested in macrophages as a measure of bioactivity following drug release incubation times, up to 14 days. Rebamipide release from brushite occurrs via non-fickian diffusion, with a rapid linear release of 9.70% ±0.37% of drug per day for the first 5 days, and an average of 0.5%-1% per day thereafter for 30 days. Rebamipide slows the initial and final cement setting time by up to 3 and 1 minute, respectively, but does not significantly reduce the mechanical strength below 4% (weight percentage). Pre-osteoblast proliferation increases by 24% upon exposure to 0.4uM Rebamipide, and by up to 73% when Rebamipide is delivered via brushite cement. Low doses of Rebamipide do not adversely affect peak alkaline phosphatase activity in differentiating pre-osteoblasts. Rebamipide weakly stimulates proliferation in macrophages at low concentrations (118 ±7.4% at 1uM), and quenches ROS by 40-60%. This is the first investigation of Rebamipide in osteoblasts.

## Introduction

In 2004 alone musculoskeletal conditions cost the US approximately 849 billion dollars[1, 2]. Out of all musculoskeletal surgical procedures in the US, half involve the graft of donor or cadaveric bone[2, 3] However, graft treatments are limited by the availability of tissue, donor morbidity, and the potential for disease transmission associated with transplanted tissue. Injectable bone substitutes, such as calcium phosphate cements (CPC), offer many advantages compared to bone grafts. Perhaps the most important feature of CPC’s is that they are very similar in composition to the mineral component of bone, and thus can precipitate new bone growth onto the cement surface (osteoconductive)[4]. The physical properties of CPC’s are also easily tuned: the mechanical strength depends upon the particle size, final porosity or stoichiometric ratio of the reactants[5, 6]. and the porosity can be controlled by varying the liquid to powder ratio, amount of crystal growth retardants or adding porosity agents such as foams or porogens.[7–9] In addition, CPC’s are also effective drug delivery vehicles[10, 11]. Delivery of bone morphogenetic protein (BMP-2), prostaglandin E2 (PGE2) receptor agonist, or vascular endothelial growth factor (VEGF) from calcium phosphates, in vivo, has been shown to yield significant increase in bone mass, bone density, and new bone surface area compared to controls[12–14]. It should be noted that dicalcum phosphate dihydrate (DCPD also known as brushite) is acidic during and after the setting reaction and, thus may better suited for small molecule drugs and not labile biological agents [15, 16].

Unfortunately, inorganic calcium phosphate cements lack many of the bioactive factors that are found in normal and demineralized bone grafts, such as BMP-2 and collagen[4]. While many studies have successfully improved bone regrowth by delivering various therapeutic agents, only BMP-2 is widely used clinically. Most biological agents are expensive to purify, even in small quantities, and stimulation of new bone growth requires large amounts[17]. Furthermore, it is difficult to deliver biological agents locally because the processing and sterilization procedures necessary for FDA approval of biomedical devices can reduce or destroy bioactivity[18]. Therapeutic agents that stimulate bone anabolism may also exert unintended effects: if local concentration is higher than expected, in other cell types, or contradictory responses within a single cell type[19]. For example, there are four receptors for PGE2 (PGE2-R1, 2, 3, 4), each with distinct action. An agent that stimulates PGE-2 production via cyclooxygenase-2 (COX-2) may increase proliferation by activating PGE receptors R1 or R2 in osteoblasts, or reduce proliferation and enhance differentiation while by activating PGE-R4[20–23].

Rebamipide is a modified hydroxyquinoline capable of reducing inflammation, enhancing regeneration, angiogenesis, mucin and proteoglycan production, and reducing cartilage degeneration, by altering the expression of genes such as COX-2, BMP-2, VEGF, hypoxia inducible factor (HIF) and PGE2-R1[24–26]. Osteoblasts are the main producer of VEGF and PGE2 during wound repair in bone, and a primary source of angiogenesis signaling following device implantation[27, 28]. Since Rebamipide is an effective stimulator of COX-2 in other cell types, we hypothesized that Rebamipide may enhance the expression of therapeutic targets COX-2, BMP-2 and/or VEGF in osteoblasts. If Rebamipide stimulates BMP-2 or PGE-2 production in osteoblasts it may be a suitable alternative to avoid the short half-life, high cost and side effects associated with other biological agents. In the present study the effect of Rebamipide on brushite cement setting, drug release and on the proliferation and differentiation of osteoblasts was investigated in the murine MC3T3 preosteoblastic cell line.

## Results

### Phase Characterization

**Fig 1:**
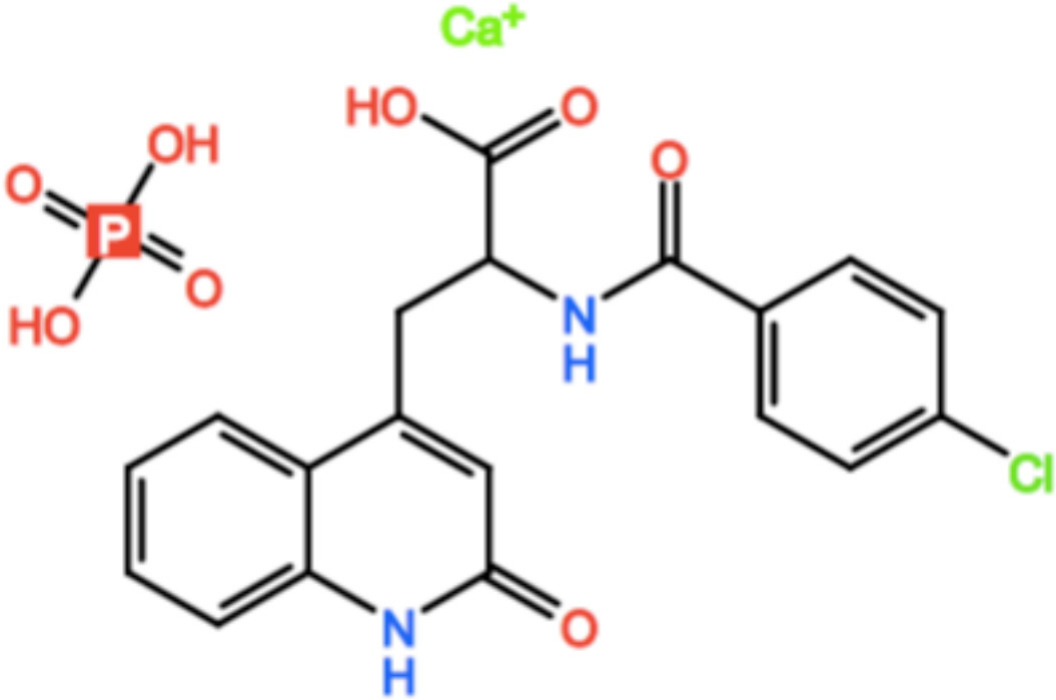
Chemical structure of Rebamipide.

**Fig 2:**
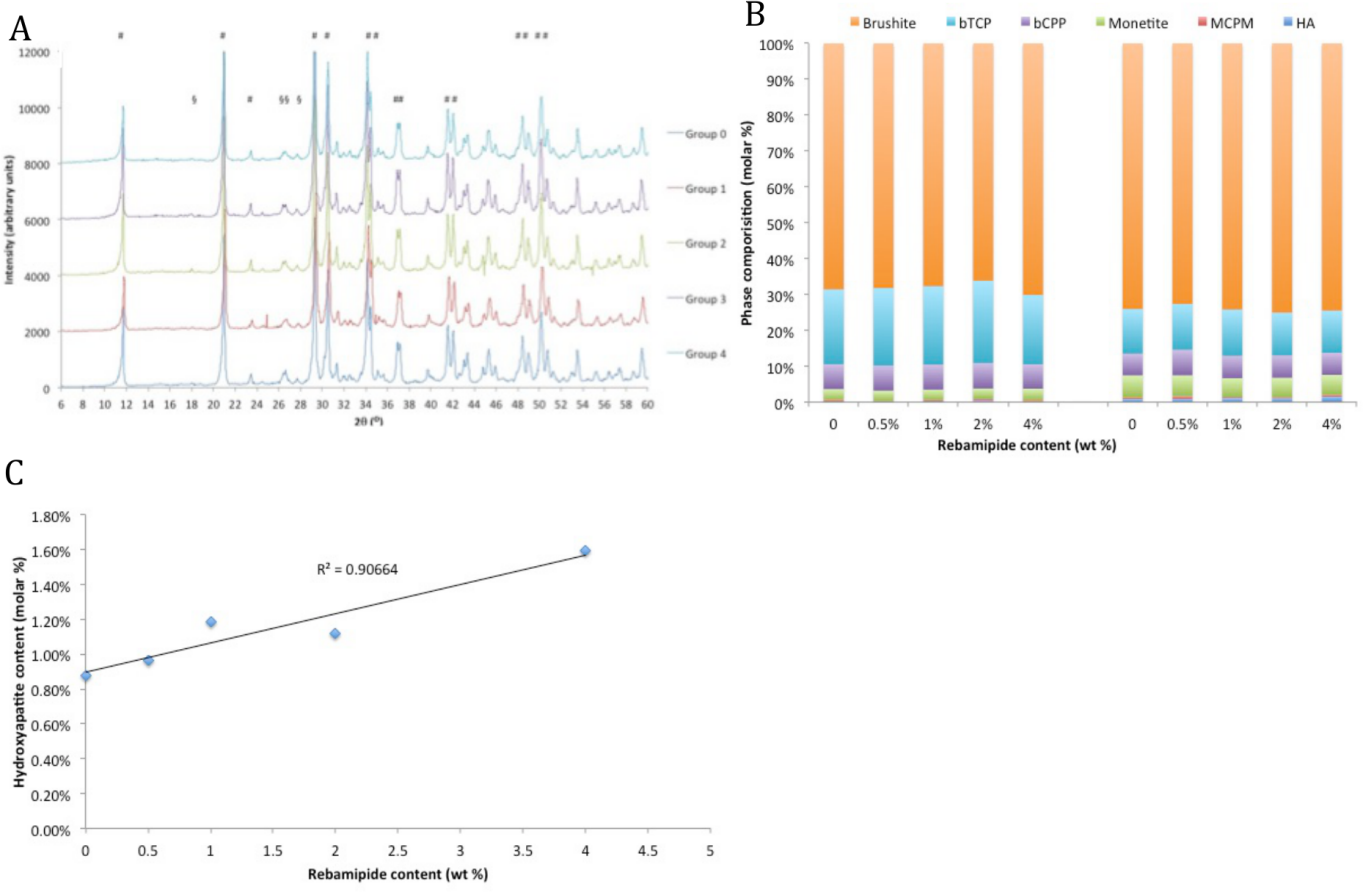
Cement phase composition. XRD diffraction spectra indicate that brushite cements containing Rebamipide are identical to unloaded cements (A) (# brushite, § bTCP, + HA). The phase composition is comparable in freshly made cements (B, left bars). The amount of hydroxyapatite in aged (30 day) composites increases with increasing amounts of Rebamipide (B, right bars, and C).

The chemical structure of Rebamipide is indicated in Fig 1. The x-ray diffraction (XRD) patterns in Fig 2A indicate that the initial phase composition of set composites did not change with increasing amounts of Rebamipide. The identified peaks all correspond to brushite or beta tricalcium phosphate (b-TCP). When the phase composition was quantitated with Rietveld refinement (Fig 2B) no difference was detected in the quantity of unreacted starting material MCPM (0.40 ±0.00%) and bTCP (21.32 ±1.36%), or initial product brushite (68.02 ±1.44%), between any of the groups. We observed a correlation between hydroxylapatite formation and Rebamipide content, in samples that had been aged for 30 days in phosphate buffered saline (PBS) (R^2^ = 0.906, Fig 2C).

### Setting Time

The initial and final setting time increased for all drug loaded composites, with an average setting time of 13.35 ±0.99 minutes for group 1–4, compared to 11.15 ±0.13 for group 0. The difference in setting time was statistically significant only for groups 2 and 4 (p = 0.006, <0.001 respectively, Tukey HSD). The average final setting time for drug loaded composites was 18.15 ±1.82 minutes, compared to 18.15 ±0.13 minutes for unloaded control cement. Group 1, and 2 composites reached final setting at 19.4 minutes, while group 3 composites reach final setting at 15.57 ±0.95 minutes.

### Mechanical Strength

**Fig 3:**
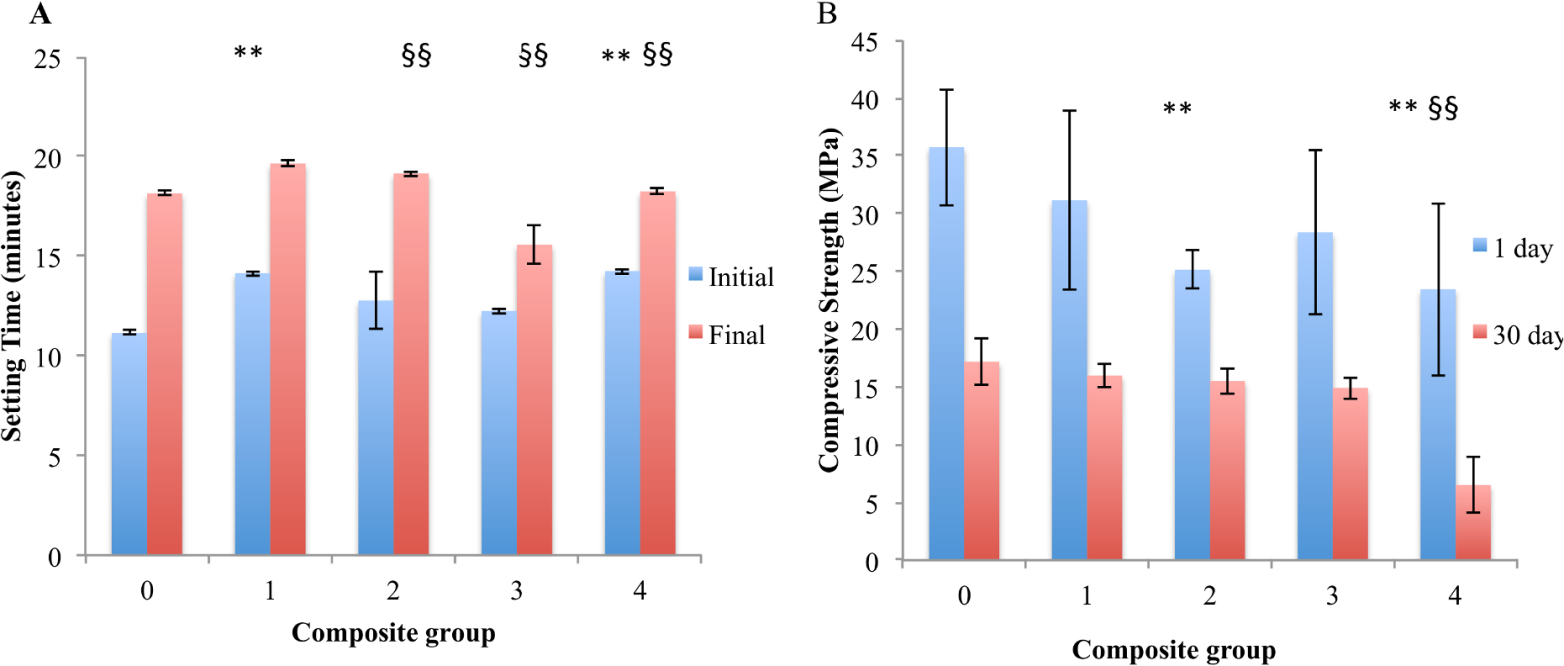
Physical properties of cements. The setting time (A) does not change with increasing amounts of Rebamipide, however the compressive strength (B) is significantly reduced in composites containing the largest amount of Rebamipide. (*, § indicate p< 0.05, **, §§ indicate P<0.01, Dunnet’s t-test).

The incorporation of a drug into cement can affect the crystal microstructure, potentially leading to a reduction in mechanical strength [16]. We investigated this possibility in drug-loaded cements after soaking in PBS for 1 or 30 days (Fig 3B). After 1 day of soaking in PBS there was a weak trend of reduced wet compressive strength with increasing Rebamipide concentrations (R^2^ = 0.626). Composites containing up to 2% Rebamipide were up to 20% weaker than controls (28MPa), though this difference was not statistically significant. Composites containing 4% Rebamipide were 35% weaker than controls. After ageing in PBS for 30 days we observed a correlation between Rebamipide concentrations and a reduction in compressive strength (R^2^ = 0.916). The average compressive strength of control cements decreased 50%, from 35MPa to 17MPa, after ageing 30 days in PBS. Aged composites containing up to 2% Rebamipide were 7–15% weaker than controls (14.7MPa) while composites containing 4% Rebamipide were 65% weaker than controls (6.5MPa).

### Drug Release

**Fig 4:**
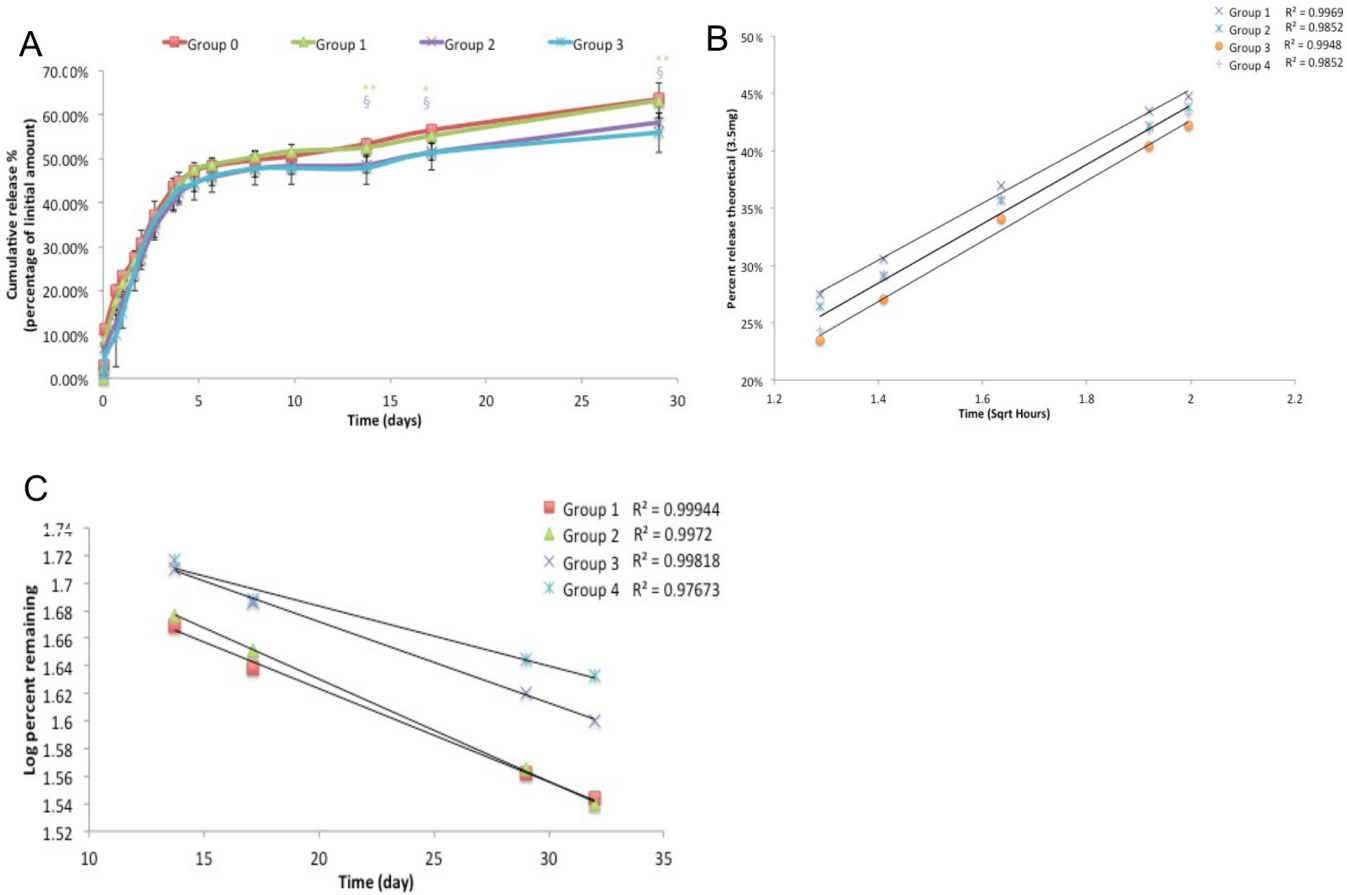
Drug release profile of cements. The release profile of cylindrical composites in PBS, over 30 days (A) (*, § indicate p< 0.05, **, §§ indicate P<0.01, Dunnet’s t-test). The first 4 days, or 45%, of release plotted using the Higuchi model (B). After 14 days the release rate closely matched first order release (C).

All of the composite formulations exhibited a rapid, linear release profile during the first 5 days (Fig 4A). During this time an average of 9.70% ±0.37% of the loaded Rebamipide was released every 24 hours in every composite groups, though this rate slowed slightly between days 3 and 5. Between 5 and 14 days the release rate plateaued, with only 3–6% of release occurring (< 1% release per day) in all composites. Also, during this time the rate of drug release began to differ based upon the absolute amount of Rebamipide remaining in the composite. Composites with 0.5% and 1% Rebamipide (group 1, 2) exhibited significantly greater (faster) cumulative drug release than 2% and 4% composites (Group 3, 4), between day 14 and day 30 (anova p<0.007, Tukey HSD p<0.05). After 14 days, all composites exhibited a second linear release phase with approximately 0.7–1% of drug released per day. The initial release phase closely matched the Higuchi model of diffusion based release (Fig 4B), while the later phase of release (after 14 days) very closely matched first order release (Fig 4C) (concentration dependent). When the logarithm of the percentage of drug released was plotted against the log of time, the slope (Korsmeyers-Peppas exponent n) indicated that all composites displayed non-fickian release rates (Table 1) (0.45 < n < 1.0). Composites with greater Rebamipide content appeared to have a larger exponent, though there is not a linear trend.

**Table 1:** Physical and drug release characteristics of Rebamipide loaded brushite cements.

Group	Drug amount (wt %)	Initial setting time (min) ±SD	Final setting time (min) ±SD	Initial strength (MPa) ±SD	Final strength (MPa) ±SD	D0-D5 Release rate (%/hour) ±SD	D14-D30 Release rate (%/hour) ±SD	Higuchi	Korsemeyers Peppas n r		First order
0	0%	11.2 ± 0.1	18.15 ± 0.1	35.75 ± 5.04	17.18 ± 2.06	-	-	-	-	-	-
1	0.5%	14.2 ± 0.1	19.65 ± 0.1	31.15 ± 7.81	15.98 ± 1.01	0.403 ±. 004%	0.027 ±. 001%	0.9969	0.56	0.9966	0.9994
2	1%	12.8 ± 1.4	19.15 ± 0.1	25.14 ± 1.61	15.51 ± 1.10	0.406 ±. 006%	0.028 ±. 001%	0.9852	0.58	0.9969	0.9972
3	2%	12.3 ± 0.1	15.6 ± 1.0	28.39 ± 7.10	14.74 ± 0.57	0.381 ±. 007%	0.025 ±. 001%	0.9948	0.67	0.9924	0.9982
4	4%	14.2 ± 0.1	18.25 ± 0.1	23.45 ± 7.48	6.51 ± 2.49	0.383 ±. 014%	0.021 ±. 001%	0.9852	0.65	0.9801	0.9767

### In Vitro Assays

#### Viability/Proliferation Test

**Fig 5:**
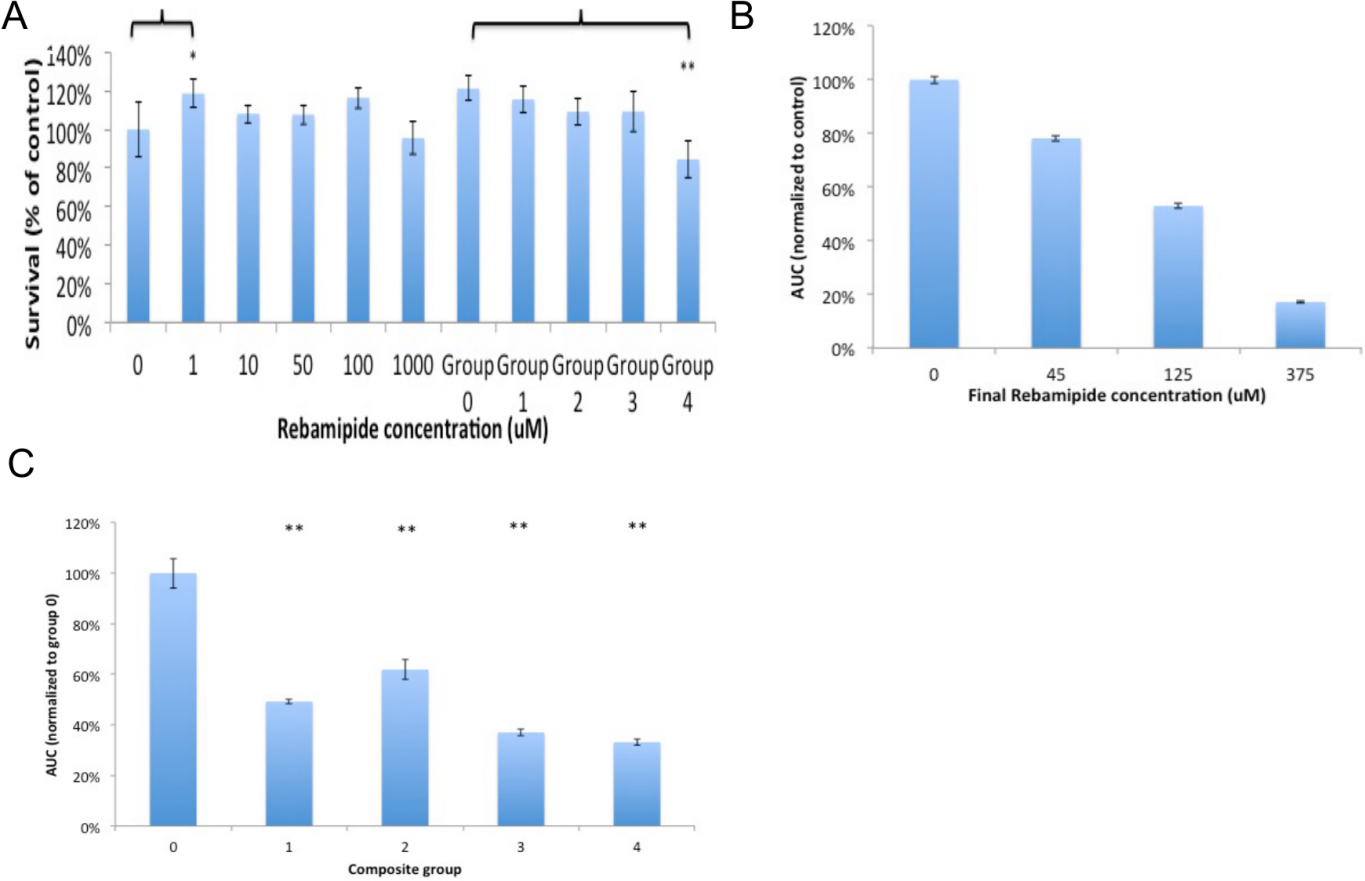
Macrophage viability and ROS production. Rebamipide does not significantly reduce the viability of macrophages up to 1mM (A). The leach product from group 4 composites was slightly toxic, but the difference in viability, compared to controls, was not statistically significant. Rebamipide quenches ROS in a dose dependent manner (B), with an estimated EC50 of 400uM in Raw 264.7 macrophages. Rebamipide, present in the leach from each composite group, was an effective quencher of ROS (C). (* indicate p< 0.05, ** indicate P<0.01 when compared to untreated, or group 0 for leach, Dunnet’s t-test).

Rebamipide stimulated proliferation of Raw 264.7 macrophages at low concentrations (1uM, 118 ±7.4% proliferation), but was otherwise comparable to controls up to 1mM (Fig 5A), indicating that Rebamipide is not toxic in macrophages even at high concentrations. Macrophages exposed to cement leach (group 0–3) exhibited an increase in proliferation (110–120% of controls), with unloaded controls (group 0) proliferating the fastest (121 ±6%). The leach from group 4 composites induced slight toxicity compared to control (84 ±9%) and a significant reduction in proliferation compared to group 0 leach (70 ± 7%).

**Fig 6:**
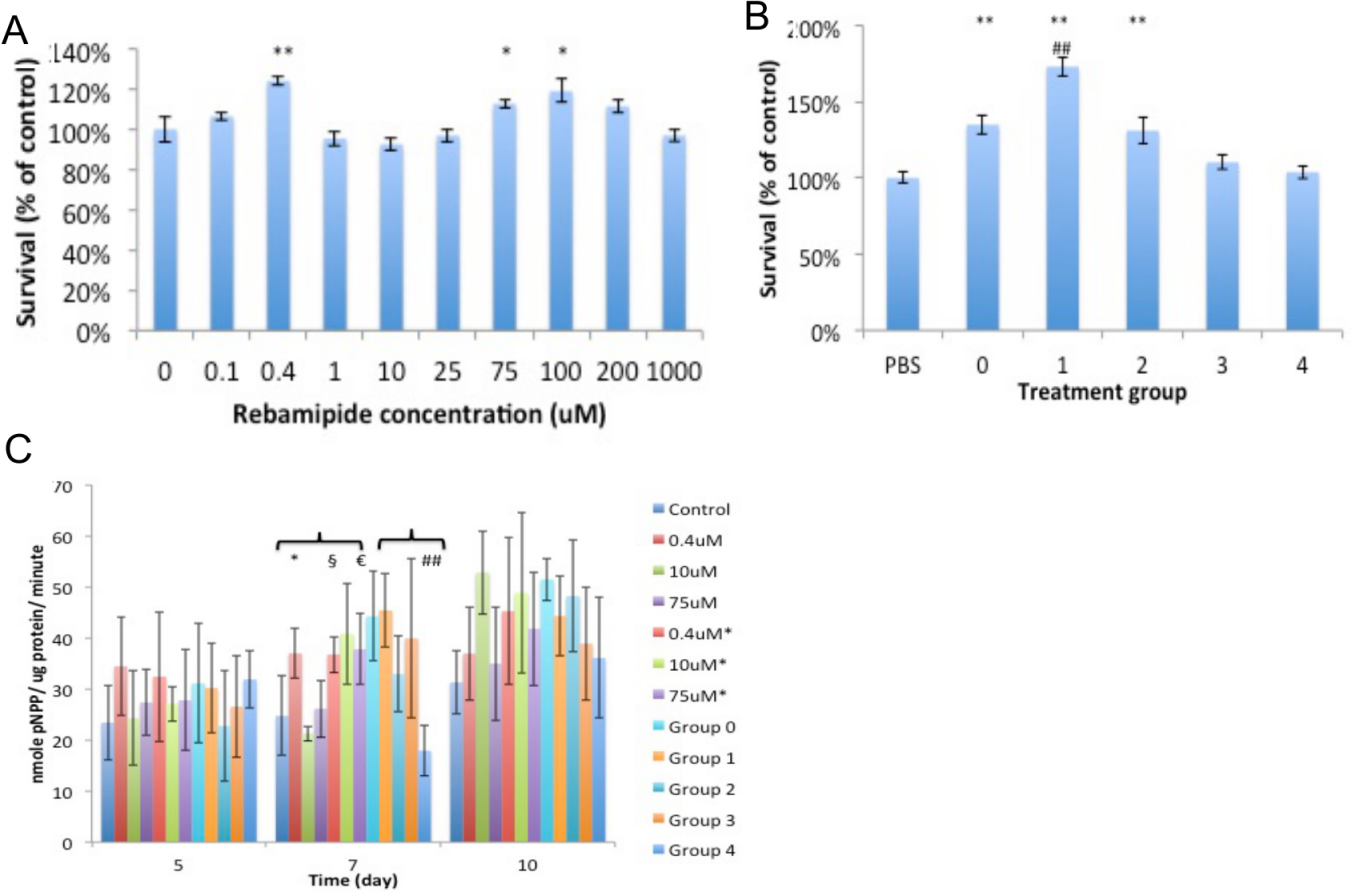
Osteoblast proliferation and differentiation. Rebamipide stimulates proliferation of osteoblasts at 0.4uM after acute (24 hours) exposure (A) (* and # indicate p< 0.05, ** and ## indicate P<0.01 when compared to untreated control, or group 0 for leach respectively, Dunnet’s t-test). The leach from control cements and Rebamipide loaded cements stimulated proliferation (B) (** indicate P<0.01 when compared to untreated control, ## indicate P<0.01 when compared to group 0 leach, Dunnet’s t-test). In differentiating osteoblasts Rebamipide exposure did not affect ALP activity, except at day 5 in 0.4uM treated samples (C) (+ (0.4uM), @ (10uM), § (0.4uM*), € (75*uM) indicate p< 0.05 compared to control, ## (group 5) indicate P<0.01 when compared to group 0 leach, Dunnet’s t-test). In the legend, concentrations with * indicate that the same concentration of Rebamipide was added at every media replacement (constant).

MC3T3 preosteoblasts exposed to 0.4uM Rebamipide for 24 hours responded with a 24% (p<0.001, Dunnet’s t-test) increase in proliferation (Fig 6A). We consistently observed a trend of increased proliferation in the 25-100uM concentration range, however statistical significance was reached in only one of three replicate experiments. High concentrations of Rebamipide (1mM) did not reduce proliferation after 24 hour exposure. When the leach products were diluted 10-fold a stimulatory effect was seen in all leaches except group 4. The unloaded leach (group 0) stimulated a 34% increase in cell number compared to osteoblasts that were not exposed to leach (PBS treated). Leach from composites loaded with the lowest amount of Rebamipide (group 1) further stimulated osteoblast proliferation by 28% compared to group 0, or by 73 ±6% compared to osteoblasts that were not exposed to leach. As the amount of Rebamipide loaded increased, the proliferation decreased, with group 4 leach matching the proliferation rate of untreated PBS controls (23% reduction in proliferation compared to unloaded cement leach from group 0) (Fig 6B). The observed reduction in proliferation occurs in a non-linear fashion. Group 2 leach has twice the Rebamipide of group 1, and group 3 has twice the concentration of group 2, etc. However, despite this linear increase in concentration group 2 leach samples proliferation 25% less than group 1, group 3 leach samples proliferate 15% less than group 2, and group 4 leach samples proliferate 5% less than group 3.

#### Antioxidant Capacity Test

Chemiluminescence assays served as a measure of the bioactivity of Rebamipide after prolonged incubation in composite/solution, to confirm that Rebamipide was not chemically altered by the mixing or setting process. Rebamipide reduced the total luminescence produced by macrophages by 50% at approximately 400uM (Fig 5B), similar to what has been reported in human neutrophils[29]. The leach products from composites reduced luminescence by 50–60% (Fig 5C).

#### Osteoblast Differentiation (ALP activity)

Alkaline phosphatase (ALP) expression is a widely recognized indicator of differentiation into mature osteoblasts. Overall, ALP activity was consistently increased in osteoblasts exposed to 0.4uM or 10uM Rebamipide, and to leach from groups 0, 1 and 2 composites (Fig 6C). During the early phase of differentiation (day 5) osteoblasts treated with 0.4uM Rebamipide consistently produced the highest ALP values of all groups (153 ±8% of untreated controls, average of 10 samples/ two experiments). By day 10 the 0.4, 10, 0.4* and 10*uM treatments produced greater ALP (167%-276% of control). Leach from group 0, 1 and 2 composites consistently stimulated greater (136–220% of untreated control) ALP activity at 7 and 10 days. Leach from group 3–4 stimulated less ALP than group 0 leach, with group 4 leach also producing less ALP than the PBS treated control group.

## Discussion

In this study we have demonstrated for the first time that Rebamipide affects the proliferation and ALP activity in MC3T3 osteoblasts. Rebamipide was a potent stimulator of proliferation at 0.4uM. The effective concentration in osteoblasts (0.4uM) and macrophages (1uM) that stimulated proliferation was quite similar. Leach containing Rebamipide exhibits an additive effect, resulting in a 73% increase in proliferation at low concentrations, but no additional enhancement in ALP activity compared to control composite leach (group 0). However, the leach from drug loaded composites appears to reduce proliferation with higher Rebamipide concentrations (Group 2–4). This biphasic effect also occurs in models of prostaglandin (PGE-2) stimulation, providing indirect evidence that Rebamipide stimulates PGE production in osteoblasts[30]. The sigmoidal (non-linear) reduction in proliferation that approaches baseline (untreated), suggest that Rebamipide is not increasingly toxic at higher concentrations. Instead the trend suggests that at low concentrations Rebamipide acts in concert with cement leach to increase proliferation, but reduces proliferation at higher concentrations by interfering with the stimulatory effects of cement leach. Perhaps most interesting, Rebamipide concentrations in the range of 0.4-10uM stimulated ALP activity, though not when delivered via brushite. For all concentrations tested Rebamipide did not appear to reduce peak alkaline phosphatase activity, suggesting it does not inhibit differentiation up to 75uM. Leach from control brushite cement stimulated proliferation and ALP, which was not surprising since calcium and phosphate ions found in the leach are well known inductors of osteoblast proliferation and differentiation[31, 32]. We propose that, much like in the case of proliferation, low concentrations of Rebamipide weakly stimulate ALP activity in MC3T3 osteoblasts while higher concentrations reduce the beneficial effects of cement leach on ALP activity. Brushite cement leach (no drug) increased ALP activity well above control and optimal Rebamipide treatments (0.4 uM). At low concentrations (group 1 composites) calcium and Rebamipide, both present in the leach, may stimulate COX-2 and PGE2 production leading to increased ALP activity. The primary bioactive component of brushite leach, calcium, is known to increase the same target of Rebamipide treatment, COX-2 and PGE2, in osteoblasts[33]. However, at higher concentrations (composite groups 2–4) Rebamipide may hinder differentiation in osteoblasts via a mechanism that is antagonistic to brushite leach. Work done by Ishihara has revealed that Rebamipide can block the activation of L-type voltage gated calcium channels, thereby affecting extracellular calcium entry[34, 35]. If Rebamipide blocks L-type calcium channels in osteoblasts it may prevent or reduce the influx of calcium from the leach, thus reducing the beneficial effects of calcium present in brushite leach [31, 36]. Though higher concentrations (10uM*, 75uM*) of Rebamipide (no leach) resulted in enhanced differentiation at 7 and 10 days, these latter treatment groups were lowered dosage regimens. By the second feeding (day 4) the delivered concentration was already 1/8 of the initial dose (i.e. the cells were treated with 75uM, 37.5uM two days and 9.375uM four days later).

Brushite is an attractive vehicle for osseous drug delivery because it is a metastable calcium phosphate, with a rapid resorption rate, that will eventually transform into either octacalcium phosphate or hydroxyapatite, in vivo[37]. Rebamipide slowed the average initial cement setting times by 1–3 minutes, but did not significantly affect the final setting time, or reduce the mechanical strength of fresh or aged samples when it was added, up to 2% of the final composite weight. The observed in setting time suggests that Rebamipide does not significantly affect the setting reaction. The delay in initial setting time is within the deviation normally found for brushite cement setting times (1–5 minutes)[8]. The initial setting strength of composites with Rebamipide were significantly stronger than the reported compressive strength (1–10 MPa) of human cancellous bone, even at the highest amount of Rebamipide [38, 39]. The observed reduction in compressive strength (50%), after ageing in PBS for 30 days, is in agreement with other studies, as brushite undergoes dissolution in PBS[39]. The phase composition of set cements was comparable between all groups. Altogether these results suggest that the Rebamipide can be added to brushite cements with minimal impact on the physical characteristics of the cement, up to 2% by weight. Though there appears to be a trend of increased hydroxylapatite formation with increased Rebamipide incorporation, the observed values are at the limit of the author’s Rietveld refinement accuracy (1–5%). Future studies should be done to determine the hydroxylapatite content after longer ageing times, perhaps after 60 or 120 days.

A cement was selected as a drug carrier because localized delivery, only at the site of a bone defect, can avoid the potential side effects associated with systemic delivery. When Rebamipide is delivered by brushite two distinct phases of release occur. The initial release is rapid, linear and predictable though it occurs via a non-fickian mechanism (0.5 < n < 1.0)[40, 41]. During the later phase of release (after 14 days) composites with higher concentrations (group 3,4) release slightly less Rebamipide than expected, as indicated by the plot in Fig 4B. Prior studies have reported that drug release occurs from brushite carriers via first-order kinetics for highly water soluble drugs[42–44]. However, Rebamipide is poorly soluble in aqueous solutions, so drug diffusion is limited by the dissolution rate (the dissolution rate has not been reported)[45]. As Rebamipide nearest to the composite surface is depleted and the diffusion layer moves further into the composite, dissolution and diffusion factors may no longer be equivalent between composite groups. Composites containing more poorly soluble Rebamipide will have less water volume, which may affect both diffusion and dissolution. In addition, while the initial amount of Rebamipide in all composites exceed the solubility, this may change after 14 days. Though the effect of Rebamipide on solution viscosity has not been investigated, as the diffusion layer extends millimeters into the composite high concentrations of Rebamipide in group 3 and 4 composites may cause small differences in viscosity that change the rate of diffusion[46]. It is important to note that the later release phase is still linear and predictable, and thus the composite continues to function as an effective drug delivery device throughout the 30 day period. Our results are in agreement with prior studies that have reported that calcium phosphate cements can continually delivery therapeutic agents in vitro, and in vivo, for more than 30 days[47].

Rebamipide has already been described as a potent anti-inflammatory and antioxidant in human macrophages and neutrophils[29, 48, 49]. In bone tissue macrophages are an integral part of the initial inflammatory response after trauma, for example after a biomaterial such as brushite is implanted. Activated macrophages react to injury or infection by releasing ROS. While ROS is necessary for wound healing, it is also directly correlated with the development of fibrotic tissue, which hampers tissue-biomaterial integration and drug release from a biomaterial[50]. Thus we examined whether Rebamipide was toxic to macrophages at concentrations where it was an effective quencher of ROS in RAW macrophages. Rebamipide was not significantly toxic to macrophages up 1mM, or when delivered via 10-fold dilutions of leach from composites (up to 300uM). The leach products (diluted 4-fold in luminescence assays) from all composite groups were effective quenchers of ROS. However, despite an approximate 8-fold increase in concentration, there was only a minimal (10%) improvement in quenching ability with greater drug loading. The leach from group 1 composites reduced ROS by the same percentage as Rebamipide diluted from the powder stock, which suggests that the bioactivity of Rebamipide is not reduced by loading it into brushite cements. However, higher doses appear to be more effective at quenching ROS when dissolved directly into solution, than when they are released from cements.

The most interesting finding of this study is that 0.4uM Rebamipide can stimulate both proliferation and differentiation. Other studies have reported an increase in gastric cell proliferation, however the proliferative effects of Rebamipide in osteoblasts has not been investigated[24, 51]. In the present study the leach containing Rebamipide was comparable in efficacy to other drugs that have been reported to stimulate osteoblast proliferation [52–59]. The optimal concentration of Rebamipide, when loaded into a brushite cement, occurs at lower loading amounts (0.5% by weight). However further testing is needed to identify whether bioactivity declines at longer elution times and, therefore, whether lower drug loading would be more effective for acute delivery. Future studies should investigate whether the increased proliferation observed at 0.4uM Rebamipide is due to an increase in PGE-2 or COX-2 production. Though Rebamipide is known to alter gene expression epithelial and chondrogenic cell types, reduce cytokine production in macrophages and neutrophils, and inhibit cancer cell proliferation, the mechanism behind each of these disparate actions is poorly understood[58].

## Conclusion

We report for the first time that Rebamipide is an effective stimulator of osteoblast proliferation and can affect ALP activity in MC3T3 osteoblasts. Rebamipide was even more effective at stimulating of proliferation when it was delivered from a brushite cement, though it no longer affected ALP expression in osteoblasts. Brushite appears to be an effective vehicle for long term (30 days) Rebamipide delivery. Osteoblast proliferation is a critical first step in the generation of new bone, and our results indicate that Rebamipide warrants further study as bone stimulating agent. Further research is needed to investigate whether BMP-2, VEGF and COX-2 expression is altered in osteoblasts exposed to Rebamipide, and whether the drug release rate from cements remain the same in biological fluids such as simulated body fluid (SBF) or blood.

## Materials and Methods

### Materials and Cell culture

All reagents and HPLC solvents, unless indicated otherwise, were purchased from Sigma-Aldrich (St. Louis, MO). Monocalcium phosphate monohydrate (MCPM) was purchased from Scharlau (Barcelona, Spain). Citric acid was purchased from Amresco (Solon, OH). Rebamipide hydrate was purchased from Santa Cruz biotechnology (Santa Cruz, CA). MC3T3 mouse calivarial osteoblasts and Raw 264.7 mouse macrophages were purchased from American Type Cell Culture (ATCC, Manassas, VA)[59, 60]. Raw cells were sub-cultured in Dulbecco’s modified eagles medium (DMEM/F12) with 10% heat inactivated fetal bovine serum (Fisher Scientific, Pittsburg, PA), 1 mg ml^-1^ streptomyocin and 1,000 units ml^-1^ of penicillin (Fisher), in 5% CO2 atmosphere at 37^°^C. MC3T3 and Raw 264.7 were subcultured upon reaching 80% confluence and only fractions containing >95% viability (trypan blue) were used for further testing. MC3T3 were tested within 8 passages of receipt from ATCC for differentiation and proliferation testing. Raw macrophages were used within 16 passages of receipt from ATCC for proliferation and ROS testing. MC3T3 cells were grown in alpha MEM medium (Gibco BRL) for subculture and proliferation studies. For differentiation studies 10mM beta-glycerol phosphate and 50ug ml^-1^ ascorbic acid was added to the proliferation media. Alkaline phosphatase substrate and microBCA kits were purchased from Sigma.

### Composite Preparation

Brushite cement was prepared by mixing MCPM and beta-TCP at 0.45:0.55 (wt %), with 0.1% (wt %) sodium pyrophosphate and 2.1% (wt %) citric acid, at a liquid to powder ratio of 0.22 ml g^-1^. MCPM particles were sieved to 75 microns in diameter to maximize the solubility, as previously described.^4^ Rebamipide powder was mixed directly into the brushite powder in a rotary mixer for 30 minutes at 4%, 2%, 1%, 0.5% or 0% (controls) of total cement mass (wt %).

### Phase Characterization

The phase composition of each group was identified by X-ray diffraction (XRD) in a Bruker D8 AXS diffractometer equipped with Cu-k0α, and Diffrac suite software provided by the manufacturer. Diffractograms were recorded on a theta-theta setup with Ni-filtered radiation, with a step size of 0.05°, from 5-60^o^ (2θ) and a step time of 2 seconds. The. raw data files were analyzed with open source Rietveld refinement software PROFEX, and compared with the profiles of beta-calcium pyrophosphate (b-CPP, PDF# 04-009-3876), bTCP (PDF# 04-008-8714), brushite (PDF# 04-013-3344), monetite (PDF# 04-009-3755), hydroxylapatite (PDF# 01-074-0565), and MCPM (PDF# 04-011-3010). The reported values are the average of three independent samples.

### Setting Time

The initial and final setting time was measured according to ASTM C266-04, with a Gilmore needle and the following modifications: stainless steel molds were used to create 4 cement discs 6mm in diameter and 2mm thick. Samples were considered set when an 113 gram load with a needle tip diameter of 2.12mm (initial setting), or a 453 gram load with a needle tip diameter of 1.06 mm (final setting), did not make a complete visible impression on the surface of the sample. For each group 1 gram of cement was mixed with water, cast within 5 minutes onto wax paper, and incubated at 37^°^C in PBS. Each sample was examined every 5 minutes and setting time was recorded.

### Mechanical Testing

Cylindrical cement samples were allowed to set in 6mmx12mm rubber molds, in phosphate buffered saline (PBS) at 37^o^ C for 24 hours. Samples were tested after 24 hours, or after ageing for 30 days in PBS. The flat surface of each cylinder was polished prior to compression testing. Each sample was tested while wet, in a Shimadzu autograph AGS-X series machine at a crosshead speed of 1 mm minute, until failure. The ultimate compressive strength was analyzed with the manufacturer supplied software, Trapezium light X. Each data point represents the average of five samples.

### Drug Release

Cylindrical samples were fabricated in rubber molds as described for mechanical testing and, after setting for 2 hours in PBS, removed from the mold and submerged in 10ml of PBS at 37^°^C. Samples were incubated at 37^°^C on an orbital platform shaker, at a constant shaking rate of 20 rpm. At predetermined time points the PBS was completely replaced with an equal volume of fresh PBS. The quantity of Rebamipide in each leach solution was analyzed with high performance liquid chromatography (Waters 2695) according to previously published methods[61]. The mobile phase was composed of 33.25% acetonitrile, 64.75% water, 1% acetic acid and 1% methanol. The retention time for integration, on a YMC Triart C18 column (50x0.2mm, 3um particle and 120Å pore size), was 2.95 ± 0.2 minutes. Each sample was run for 7 minutes, at a flow rate of 0.15 ml min^-1^, with an injection volume of 5uL and detected with a Waters 2996 UV lamp (246nm). The Empower 2vA6.2 software provided by the manufacturer was used to identify and integrate the area under the curve. The release profile was fit to Higuchi and Korsmeyer-Peppas during the first 60% of release, and to a first order release profile during the later phase of release. Only data points after the first 24 hours of release were fit to ensure the cement composites had set.

For subsequent in vitro testing leach was collected between day 3 and 10 for ROS, or day 10 and 20 for viability and differentiation, yielding solutions containing approximately 10% and 5% of total loaded drug in a 10ml volume, respectively. For ALP studies these concentrated stocks were then diluted over the course of 14 days, as described below (5.7.3). Three samples from each group were pooled, neutralized, sterile filtered, and the concentration was determined by HPLC. The typical concentration range of Rebamipide collected between day 3 and 10 was 120-200uM (group 1), 350-420uM (group 2), 650-800uM (group 3), and 1200-1400uM (group 4). Leach collected between day 10 and 20 were lower concentration: 50-90uM (group 1), 100-180uM (group 2), 200-400uM (group 3), and 750-1000uM (group 4).

### In Vitro Tests

#### Viability/Proliferation Test

Osteoblasts and macrophages are two of the most abundant and critical cell populations involved in osseous regeneration following surgical trauma, and thus were selected for bioactivity studies. Acute toxicity was determined by alamar blue reduction. Raw 264.7 cells were seeded at 8,000 cells cm^-2^ into 96 well plates and allowed to proliferate for 3 days, until 80% confluent. MC3T3 cells were seeded at 10,000 cells cm^-2^ and allowed to proliferate for 1 day before treating. At the time of treatment the media was replaced with known concentrations of Rebamipide dissolved in media, or neutralized leach from cement composites. The cement leach was diluted 10-fold for macrophage and osteoblast cultures to examine the bioactivity (proliferation) of Rebamipide after elution from a CPC. After 24 hours the media was replaced with a 5% solution of alamar blue, dissolved in proliferation media, and incubated for 1.5 hours at 37^°^C. The fluorescence of the reduced alamar blue product, Resorufin, was detected at 560 and 590nm (excitation/emission), background (no cells) was subtracted, and the values were normalized to the respective untreated controls and expressed as percent survival at each time point.

#### Antioxidant Capacity Test

The ability of Rebamipide to quench reactive oxygen species (ROS) in Raw 264.7 macrophages was determined by a luminol amplified chemiluminescence assay. 200,000 cells were suspended in 150uL of luminol buffer containing 50mM luminol, 0.1M NaOH, 2ug ml^-1^ of horse radish peroxidase, 100uM glucose and 3 parts of RPMI-1640 for every 1 part of PBS. A 50uL aliquot of PBS, a known concentration of Rebamipide, or leach solution from drug-loaded composites was added to bring the final solution volume to 200uM. All cells were activated with 1uM of phorbol myristate acetate (PMA). Cells were incubated in 96-well white optiplate (Perkin Elmer) at 37^°^C and luminescence readings (492nm) were recorded every two minutes, for 1 hour, on a Tecan plate reader. The total area under the curve was integrated and the total plotted to determine the effective quenching ability of Rebamipide. Each sample was run in triplicate.

#### Osteoblast Differentiation (ALP activity)

MC3T3 were seeded at 30,000 cells cm^-2^ for differentiation studies, in 96 well plates. After 1 day, the growth media was replaced with differentiation media and known concentrations of Rebamipide or neutralized cement leach. In vitro and in vivo the amount of drug release from composites decreases with time. Consequently, differentiating osteoblasts were treated with either a descending, or constant (indicated on the X axis of Fig 5c by a *, i.e. 0.4uM*) treatment regimen. In the constant dose regimen cells were treated with the same concentration of Rebamipide every time the media was replaced. The descending dose group received the indicated initial dose, however treatment was diluted by a factor of 2, 4, 16 and 32-fold when the media was replaced on day 2, 4, 6 and day 8–12 (i.e. the 0.4uM group was treated with 400, 200, 100, and 25nM on day 0, 2, 4 and 6 respectively, and 12.5nM on day 8, 10 and 12). The descending dose regimen mimicked the observed drug release rate in vitro. Unfortunately, because of the limited solubility of Rebamipide, the concentration of Rebamipide obtained from in vitro leach was too low to allow for further dilution into cell culture. For this reason a concentrated stock was necessary. At 5, 7 or 10 days cells were washed with PBS and lysed with 200uL of lysis buffer (20mM Tris, 1mM MgCl, 0.1mM ZnCl, 0.1% Triton-X 100) and immediately frozen at -20^°^C for later analysis. The lysates were freeze/thawed three times at 37^°^C and a 25uL aliquot was taken from each well and combined with 50uL of alkaline phosphatase substrate for 20 minutes. The reaction was stopped by adding 25uL of 3M NaOH, and the conversion of substrate p-nitrophenylphosphate into free 4-nitrophenol was determined by spectrophotometer (Tekan plate reader) at 405nm. A 50uL aliquot was taken from each well and combined with an equal volume of micro-BCA working solution, per the manufacturer’s instructions, incubated at 37^°^C for 1 hour, and the absorbance was read at 562nm on a Tekan plate reader. ALP and BCA readings were compared to a standard curve of 4NP and BSA respectively, and the calculated alkaline phosphatase activity was normalized to the protein content for each well. Experiments were replicated twice, with 5 samples per group in each experiment.

### Statistical analysis

The results of phase composition, mechanical strength, drug release and setting time experiments were analyzed with ANOVA (IMB SPSS Software) and Tukey HSD post-hoc analysis. In vitro test were analyzed with ANOVA (IMB SPSS Software) to detect differences between groups, and Dunnet’s t-test for multiple dose comparisons to a single control value.
